# Effect of Rare-Earth Doping on Free-Volume Nanostructure of Ga-Codoped Glassy (As/Sb)_2_Se_3_

**DOI:** 10.1186/s11671-017-1959-2

**Published:** 2017-03-14

**Authors:** Yaroslav Shpotyuk

**Affiliations:** 1 Department of Sensor and Semiconductor Electronics, Ivan Franko National University of Lviv, 107, Tarnavskogo str., Lviv, 79017 Ukraine; 2 Center for Innovation and Transfer of Natural Sciences and Engineering Knowledge, Faculty of Mathematics and Natural Sciences, University of Rzeszow, 1, Pigonia str., 35-959 Rzeszow, Poland; 3 Laboratoire Verres et Céramiques, UMR-CNRS 6226, Université de Rennes 1, 35042 Rennes Cedex, France

**Keywords:** Rare-earth doping, Positron annihilation lifetime spectroscopy, Atomic-deficient nanostructurization, Sb-modification

## Abstract

Subsequent stages of atomic-deficient nanostructurization finalizing rare-earth functionality under Pr^3+^-doping in Ga_2_(As_0.28_Sb_0.12_Se_0.60_)_98_ glass are studied employing method of positron annihilation lifetime spectroscopy. Genesis of free-volume positron trapping sites, composed of atomic-accessible geometrical holes (void cores) arrested by surrounding atomic-inaccessible Se-based bond-free solid angles (void shells), are disclosed for parent As_2_Se_3_, Ga-codoped Ga_2_(As_0.40_Se_0.60_)_98_, as well as Ga-codoped and Sb-modified Ga_2_(As_0.28_Sb_0.12_Se_0.60_)_98_ glasses. The finalizing nanostructurization due to Pr^3+^-doping (500 wppm) in glassy Ga_2_(As_0.28_Sb_0.12_Se_0.60_)_98_ is explained in terms of competitive contribution of changed occupancy sites available for both rare-earth ions and positrons.

## Background

Glassy-like compounds of chalcogens (i.e., S, Se, Te) with some elements from IV-V groups of the periodic table (typically Ge, As, Sb, Bi), also known as chalcogenide glasses (ChG) [1, 2], compose a promising class of functional media for modern optoelectronics and IR optics [2–5]. Because of wide transparency window up to 20 μm accompanied by low phonon absorption, good chemical durability, and glass-forming ability, the ChG provide an excellent platform for modern fiber-optic amplifiers and mid-IR lasers [4, 5].

To be functional in many of such active photonic applications, the ChG should successfully operate as high-efficient *host* matrices for embedded *guest* activators in the form of rare-earth (RE) ions (such as Dy^3+^, Er^3+^, Pr^3+^) [5]. This can be achieved by useful modification of ChG at a nanoscale level due to *nanostructurization*, the process stretching over both atomic-specific and atomic-deficient (free-volume) structural arrangement at a nanospace. From most generalized viewpoint, such nanostructurization route includes subsequent stages of glass structure modification to meet requirements of effective *charge compensator*, *devitrification inhibitor*, and *low phonon energy RE hosting site*.

In this work, at the example of glassy arsenic selenide g-As_2_Se_3_, one of most popular ChG for waveguide optical sensing, IR lasers and telecommunication [6], we shall trace evolution of atomic-deficient glass structure during these stages (*atomic-deficient* or *free-volume nanostructurization*), employing the method of positron annihilation lifetime (PAL) spectroscopy, one of most efficient tool to study free-volume elements (FVE) in different solids (like vacancies, vacancy-type clusters, voids, pores, intrinsic cracks) at atomistic and sub-atomistic length-scales [7–10].

## Methods

### Nanostructurization Technologies in Chalcogenide Photonics

Nanostructurization is aimed to ensure high-efficient chemical environment in which RE ions reside homogeneously without clustering, crystallization, and phase separation.

*The first stage* in this row of nanostructurization technologies belongs just to glass preparation owing to conventional melt-quenching route, which is described in details elsewhere [11–13].

For this research, the ChG of stoichiometric g-As_2_Se_3_ (i.e., As_40_Se_60_) were prepared from high-purity elemental precursors, e.g., As (5 N) and Se (5 N), these ingredients being specially purified by distillation with low evaporation rate to remove impurities (such as O, C, H_2_O, and SiO_2_). Appropriate amounts of ingredients with total weight close to 30 g were put into silica tube of 10 mm diameter. Then, the ampoules were sealed under a vacuum, heated up to 900 °C with 2 °C/min rate and stayed at this temperature for 10 h in a rocking furnace with further quenching into water from 700 °C. To remove mechanical strains appeared during rapid quenching, the alloys were annealed for 6 h at 10 °C less than the glass transition temperature. Then, the obtained rods were cut into ~2-mm disks and polished.

*The second stage* in nanostructurization is to prepare the ChG with locally disturbed covalent glass-forming network possessing effective charge-compensation properties for potential RE dopants. In respect to g-As_2_Se_3_-based media, this can be achieved due to doping with small amount of Ga (or alternatively, In), allowing stabilization of optimal compound with maximal Ga content, but still in glassy state [14–18]. The procedure of such Ga codoping is realized via the same melt-quenching technological route as for g-As_2_Se_3_ using high-purity elemental Ga (7 N purity). As was shown in our preliminary research [13, 17], the Ga-codoped g-As_2_Se_3_ is optimized under chemical composition of g-Ga_2_(As_0.40_Se_0.60_)_98_.

*The third stage* in nanostructurization is to modify the Ga-codoped ChG against possible parasitic devitrification (phase separation, crystallite nucleation, extraction, and growth), which can be activated in ChG under further RE doping. One of the best resolutions is transferring to partial As to Sb replacement in g-As-Se, allowing optimal Ga-codoped g-Ga_2_(As_0.28_Sb_0.12_Se_0.60_)_98_ prepared by melt-quenching route like g-As_2_Se_3_ or g-Ga_2_(As_0.40_Se_0.60_)_98_ [19].

*The fourth stage* in nanostructurization is just finalizing RE-doping technology, i.e., the process, which is also realized under conventional melt-quenching using some precursors for RE dotation, such as Pr_2_Se_3_ (3 N purity). Within row of examined glassy arsenic selenides g-As-Se, this stage results in optimal g-Ga_2_(As_0.28_Sb_0.12_Se_0.60_)_98_ affected by RE doping with 500 wppm of Pr^3+^.

### PAL Spectroscopy as Instrumentation Tool Tracing Atomic-Deficient Nanostructurization

*The PAL measurements* were performed using a fast-fast coincidence system of 230 ps resolution based on two Photonis XP2020/Q photomultiplier tubes coupled to BaF_2_ scintillator 25.4A10/2M-Q-BaF-X-N detectors (Scionix, Bunnik, Holland) and ORTEC® electronics (ORTEC, Oak Ridge, TN, USA). The reliable PAL spectra were detected in a normal-measurement statistics (~1 M coincidences) under stabilized temperature (22 °C) and relative humidity (35%). The channel width of 6.15 ps allows a total number of channels to be 8000. The radioactive ^22^Na isotope of relatively low ~50 kBq activity prepared from aqueous solution of ^22^NaCl wrapped by Kapton® foil (DuPont™, Circleville, OH, USA) of 12 μm thickness was used as positron source sandwiched between two identical tested samples.

The raw PAL spectra were processed with LT 9.0 program [20]. Under unchanged contribution from a source (with 372 ps and ~2 ns inputs), these spectra were decomposed into two normalized components with *τ*_*1,2*_ lifetimes and *I*_*1,2*_ intensities (*I*_*1*_ + *I*_*2*_ = 1). Under above spectrometer resolution, this allows an error-bar for such arranged measuring protocol to be not worse than ±0.005 ns in lifetimes and ±0.01 in intensities. Introducing third component in the envelope of fitting curves did not improve goodness of fitting significantly (the bound positron-electron states were not proper for studied Se-based ChG in full agreement with previous results [17, 18].

*The PAL response on atomic-deficient nanostructurization* of the ChG was identified within canonical two-state positron trapping (PT) model [7–10, 21, 22], assuming that *x*2-term reconstructed PAL spectrum represents only one kind of FVE. Under such circumstances, the center of mass of the reconstructed PAL spectrum coincides with average positron lifetime *τ*_*av*_ defined through *normalized fractions* of positron annihilation channels at defect-free bulk *η*_*b*_ and defect-specific *η*_*d*_ = *τ*_*1*_⋅*κ*_*d*_ states (*I*_*1*_ + *I*_*2*_ = *η*_*b*_ + *η*_*d*_ = 1):

1 $$ {\tau}_{av}={I}_1{\tau}_1+{I}_2{\tau}_2={\eta}_b{\tau}_b+{\eta}_d{\tau}_d. $$

Other physical quantities (i.e., PT-modes), in part, defect-free bulk positron lifetime *τ*_*b*_ and PT-rate in defects *κ*_*d*_, can be calculated from *x*2-term parameterized PAL spectrum as:

2 $$ {\tau}_b={\tau}_B=\frac{1}{\lambda_b}=\frac{\tau_1{\tau}_2}{I_1{\tau}_2+{I}_2{\tau}_1}, $$

3 $$ {\kappa}_d={I}_2\left(\frac{1}{\tau_1}-\frac{1}{\tau_2}\right)=\frac{I_2}{I_1}\left(\frac{1}{\tau_b}-\frac{1}{\tau_d}\right). $$

In addition, the *(τ*_*2*_ *-τ*_*b*_ *)* difference can be accepted as a size measure for extended free-volume PT sites where positrons are trapped, as well as the *τ*_*2*_ */τ*_*b*_ ratio can be taken as direct signature of nature of these PT defects in terms of equivalent number of monovacancies [7].

Within canonical two-state PT model, the PAL response on *atomic-deficient nanostructurization* is defined by changes in *defect-specific annihilation channel*, which is determinant of PT-rate in defects *κ*_*d*_ [23]. Typically, these processes are not accompanied by any changes in defect-free bulk positron lifetime *τ*_*b*_, but changes in the content and sizes of PT defects (reflected in the intensity of the second component *I*_*2*_ and defect-specific positron lifetime *τ*_*2*_) can be essential.

The most drastic nanostructurization-induced changes concern FVE disappearance (void collapse) or, contrary, FVE appearance (void creation), the disappearing (appearing) voids being fully excluded from overall PT in ChG. Within *x*2-component PAL-spectra fitting, the PT-reduction due to FVE collapse results from decreased *I*_*2*_ intensity accompanied by more slightly changed *τ*_*2*_ lifetime. However, the nanostructurization can also lead to more evolutional changes in atomic-deficient void structure, associated preferentially with nearest environment of FVE. Such evolutional changes based on mutually opposite processes of void agglomeration (fragmentation), expansion (contraction), coarsening (refining), and charging (discharging) [23] are also finished in modified PT-rate in defects *κ*_*d*_. Thus, the PT-reduction owing to *agglomeration* of relatively large voids, which get favorable environment to grow in size owing to their merge [23, 24], is accompanied by decrease in *I*_*2*_ intensity and slight increase in *τ*_*2*_ lifetime. At the same time, the PT-enhancement (i.e., increase in the PT-rate *κ*_*d*_) determines *fragmentation* of relatively large free-volume voids, which tend to be tiny owing to grinding (decaying on separate parts), this process being accompanied by increased *I*_*2*_ intensity and decreased defect-related *τ*_*2*_ lifetime.

## Results and Discussion

Let us trace evolution of atomic-deficient (free-volume) nanostructurization over four subsequent stages (parent glass—Ga-codoping—Sb-modification—RE doping) using the PAL spectroscopy data for typical samples of arsenic selenide ChG.

**Fig. 1:**
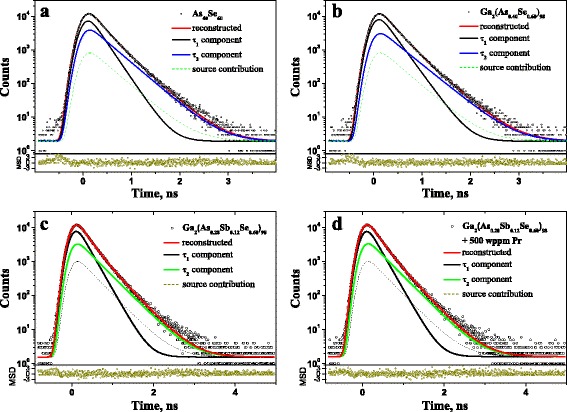
Raw PAL spectra of g-As_40_Se_60_ (**a**), g-Ga_2_(As_0.40_Se_0.60_)_98_ (**b**), g-Ga_2_(As_0.28_Sb_0.12_Se_0.60_)_98_ (**c**), and Pr^3+^-doped (500 wppm) g-Ga_2_(As_0.28_Sb_0.12_Se_0.60_)_98_ (**d**). The *bottom insets* show statistical scatter of variance

The measured raw PAL spectra are reconstructed from *x*2-term fitting procedure, these spectra for parent g-As_2_Se_3_, Ga-codoped g-Ga_2_(As_0.40_Se_0.60_)_98_, Ga-codoped and Sb-modified g-Ga_2_(As_0.28_Sb_0.12_Se_0.60_)_98_, Pr^3+^-doped (500 wppm) g-Ga_2_(As_0.28_Sb_0.12_Se_0.60_)_98_ being depicted on Fig. 1. The limited values of scatter of variance tightly grouped along 0-axis testify that PAL probing is adequately described within this fitting procedure. Therefore, decaying behavior of the PAL spectra on Fig. 1 can be reflected by sum of two negative exponentials with different time constants inversed to positron lifetimes. The best-fit positron trapping modes for the examined ChG calculated within two-state PT model [7–10, 21, 22] are given in Table 1.

**Table 1:** Fitting parameters and PT-modes describing two-component reconstructed PAL spectra in g-Ga_x_[(As/Sb)_0.40_Se_0.60_]_100-x_

ChG sample	Fitting parameters			Positron trapping modes					
	*τ*_*1*_	*τ*_*2*_	*I*_*2*_	*τ*_*av.*_	*τ*_*b*_	*κ*_*d*_	*τ*_*2*_ *-τ*_*b*_	*τ*_*2*_ */τ*_*b*_	*η*_*d*_
	ns	ns	a.u.	ns	ns	ns^−1^	Ns	–	–
g-As_40_Se_60_	0.210	0.360	0.462	0.279	0.260	0.92	0.10	1.39	0.19
g-Ga_2_(As_0.40_Se_0.60_)_98_	0.223	0.382	0.401	0.287	0.267	0.75	0.11	1.43	0.17
g-Ga_2_(As_0.28_Sb_0.12_Se_0.60_)_98_	0.210	0.363	0.422	0.274	0.255	0.85	0.11	1.42	0.18
Pr^3+^-doped (500 wppm) g-Ga_2_(As_0.28_Sb_0.12_Se_0.60_)_98_	0.218	0.374	0.376	0.276	0.258	0.72	0.12	1.45	0.16

### Free-Volume Nanostructurization in Parent g-As_2_Se_3_

The parent g-As_2_Se_3_ possesses defect-specific lifetime *τ*_*2*_ = 0.360 ns (Table 1) proper to this ChG as it follows from numerous previous research [25–28]. The positron trapping in g-As_2_Se_3_ is defined by PT-rate *κ*_*d*_ = 0.92 ns^−1^ occurring under fraction of trapped positrons *η*_*d*_ = 0.19. In respect to Jensen et al.’s DFT-calculations for orthorhombic As_2_Se_3_ crystal [27], this lifetime gives an estimate for volume of PT defects near ~90 Å^3^. This open volume corresponds to 0.10 ns in (*τ*_*2*_ *-τ*_*b*_) difference and 1.39 in *τ*_*2*_/*τ*_*b*_ ratio, which can be accepted as a signature of extended triple-quadruple vacancies [7, 27]. It is difficult to define exactly which part of this free volume is atomic-accessible in glassy network in view of complicated inner structural configuration composed of interconnected atom-shared AsSe_3/2_ pyramids in g-As_2_Se_3_. In ref. [17], possible configuration of such PT free-volume voids were depicted at the map of electron-density distribution for isostructural mineral orpiment As_2_S_3_.

**Fig. 2:**
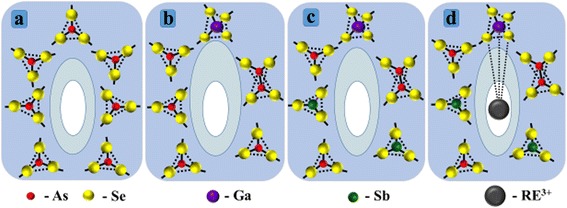
Genesis of free-volume PT-site in g-As-Se under subsequent nanostructurization stages evolving parent g-As_40_Se_60_ (**a**), Ga-codoped g-Ga_2_(As_0.40_Se_0.60_)_98_ (**b**), Ga-codoped and Sb-modified g-Ga_2_(As_0.28_Sb_0.12_Se_0.60_)_98_ (**c**), and Pr^3+^-doped (500 wppm) g-Ga_2_(As_0.28_Sb_0.12_Se_0.60_)_98_ (**d**). The inner atomic-accessible free-volume core is marked by *white color*, the outer atomic-inaccessible free-volume shell is *blue-light-shadowed*, while surrounding glassy network composed by different glass-forming polyhedrons is *blue-dark-shadowed* (see text for more details)

Structural genesis of expected PT sites in parent g-As_2_Se_3_ is conditionally illustrated on Fig. 2a assuming close to ellipsoidal shape for free-volume voids. The most efficient preferential PT sites are defined by extended free-volume spaces near Se atoms neighboring with AsSe_3/2_ polyhedrons [26, 27]. Because of strong directionality of covalent chemical bonding in ChG, Se atoms form lower electron-density spaces known as *bond-free solid angles* (BFSA) in terms of Kastner [29]. These *atomic-inaccessible BFSA* contribute to neighboring geometrical free volumes, ensuring effective negative electrical charge due to proximity with electronegative Se atoms linked with more electropositive As. So, the BFSA originated from Se atoms form *outer shell* for *inner geometrical hole* of free-volume void, which can be identified in view of its preferential electric state as counterparts of cation-type vacancy in crystals [7].

Therefore, the most efficient PT sites in g-As_2_Se_3_ can be imaged as free-volume voids formed within network of interlinked corner-shared AsSe_3/2_ pyramids, composed of atomic-accessible geometrical hole (*void core*) arrested by surrounding atomic-inaccessible Se-based BFSA (*void shell*), as it is illustrated in Fig. 2.

### Free-Volume Nanostructurization Under Ga-Codoping

Effect of Ga-codoping in parent g-As_2_Se_3_, i.e., transition from g-As_2_Se_3_ to g-Ga_2_(As_0.40_Se_0.60_)_98_, is revealed through gradual dropping in *I*_*2*_ intensity accompanied by increase in defect-specific *τ*_*2*_ lifetime to 0.382 ns (Table 1). At the basis of Jensen et al.’s [27] formalism, the latter can be ascribed to free volumes reaching as high as ~110 Å^3^. This jump in defect-specific *τ*_*2*_ lifetime is ascribed to increased average atomic coordination *Z* = 2.412 of g-Ga_2_(As_0.40_Se_0.60_)_98_ due to Ga addition. Under such condition, the appeared Se_2/2_-As-As-Se_2/2_ bridges counterbalance Ga additions in g-As-Se, causing increased number of overlapped BFSA around end-terminated Se atoms contributing to PT sites [17, 28]. Thus, the Ga-codoping in g-As_2_Se_3_ results in *agglomeration* of existing PT sites (increase in their volume, but decrease in their content), thus leading to gradual decrease in PT-rate in defects *κ*_*d*_ and, correspondingly, the fraction of trapped positrons *η*_*d*_ (see Table 1).

It is worth to note that PAL response on Ga-induced nanostructurization is fully determined by chemical composition of parent ChG. Thus, for example, in case of smaller *Z* = 2.30 character for TAS-235 glass (i.e., g-As_30_Se_50_Te_20_) [18], Ga-codoping does not change defect-specific *τ*_*2*_ lifetime, despite more pronounced decrease in *I*_*2*_ intensity. This result is fully concomitant with small deviations observed in *τ*_*2*_ lifetimes for Se-rich ChG compositions in As-Se system [26, 28].

In general, such modification (when only atomic-deficient free-volume structure is changed) is unable to accommodate RE ions obeying electrically active state (RE^3+^), avoiding non-radiative decay [5, 14, 17, 18]. The FVE-accommodated RE dopants have to adopt an excess of positive electrical charge to ensure electrical compensation throughout a glassy matrix. Successful resolution is based on possibility of Ga-codopants to reveal a metallic behavior being inserted in chalcogenide environment. In interaction with chalcogens, the Ga atoms create some polyhedrons (such as GaSe_4/2_ tetrahedra shown in the upper part on Fig. 2b), which are, from one side, topologically consistent with main network-forming polyhedrons to attain unique glassy arrangement having a large number of voids, but, from other side, these codopants can stabilize charge misbalance owing to local chalcogen (Se) over-coordination around Ga [14, 16, 30]. Under transition to g-Ga_2_(As_0.40_Se_0.60_)_98_, the GaSe_4/2_ tetrahedrons with favorable Ga-Se chemical bonds appear in a network of corner-shared AsSe_3/2_ pyramids forming overall glassy matrix. Excess of anion-type atoms occupying Se^2−^ states around GaSe_4/2_ tetrahedrons causes the cloud of preferentially negative electrical charge for neighboring free-volume void (as it is shown by enlarged outer shell in the constitution of PT site on Fig. 2b). In such a way, the more negatively charged voids of increased overall free volume in Ga-modified g-Ga_2_(As_0.40_Se_0.60_)_98_ serve as eventual precursors for *charge-compensating incorporation* of electrically active Pr^3+^ ions.

### Free-Volume Nanostructurization Under Sb-Modification

One of the parasitic drawbacks of Ga-codoping nanostructurization concerns in increased crystallization ability of Ga-contained ChG under further RE doping, since both Ga and RE chalcogenides possess isostructural crystalline polymorphs [5, 13]. In case of As-based ChG, this obstacle can be suppressed under partial As-to-Sb replacement [19]. This was a reason to turn towards nanostructurization in g-As-Se under Sb-modification.

In respect to atomic-deficient structure evidenced from PAL spectroscopy, this Sb-substituted g-Ga_2_(As_0.28_Sb_0.12_Se_0.60_)_98_ demonstrates partial recovery to parent pure g-As_2_Se_3_. Indeed, in this ChG, the defect-specific lifetime *τ*_*2*_ is depressed down to 0.363 ns and second component intensity *I*_*2*_ gets elevated to 0.422, thus resulting in steadily increasing PT-rate in defects with *κ*_*d*_ *=* 0.85 ns^−1^ (Table 1). This Sb-modification is not accompanied by change in PT-site type, since neither (*τ*_*2*_ *-τ*_*b*_) difference, nor *τ*_*2*_ */τ*_*b*_ ratio remains rather unchanged within measuring error-bar.

Such effects are supposed to be defined by increase in an atomic packing of glassy network due to heavier (and more metallic) Sb atoms appeared instead of As ones (causing respective increase in ChG density from 4.64 to 4.90 g/cm^3^ [19]). On Fig. 2c, these Sb-based structural entities are presented as SbSe_3/2_ pyramidal units appeared in the nearest atomic surrounding of free-volume void. So PT sites in Sb-modified g-Ga_2_(As_0.28_Sb_0.12_Se_0.60_)_98_ are like those in g-Ga_2_(As_0.40_Se_0.60_)_98_, but with slightly reduced outer free-volume shell mainly due to shielding effect from more metallic Sb-environment (Fig. 2c).

### Free-Volume Nanostructurization Under RE Doping

Thus, in respect to these subsequent nanostructurization stages, the effect of RE doping can be treated in terms of *competitive contribution of changed occupancy sites* in the modified structure of g-Ga_2_(As_0.28_Sb_0.12_Se_0.60_)_98_ available for both RE ions and positrons. Indeed, from the point of affinity to negative electrical charge attached to neighboring free-volume space, the same type of voids, which accommodate RE^3+^ ions, as shown in Fig. 2d, can be attractive sites for annihilating positrons [6–10]. Under RE doping, the positively charged Pr^3+^ ions are stabilized in a glassy network due to strong Pr^3+^-Se-Ga covalent bridges (marked by dotted lines in Fig. 2d) [14, 31, 32], thus eliminating corresponding negatively charged void as potential positron traps. This void-collapse process results in PT-reduction, mainly due to essential decrease in second component intensity *I*_*2*_ and rather slight increase in defect-specific lifetime *τ*_*2*_ (see Table 1), meaning that void volume is not essentially altered under RE doping.

Concentration of these PT free-volume defects in RE-doped ChG can be estimated accepting their analogy with negative cation-type vacancies in semiconductors giving trapping coefficients of approximately 10^15^ atom⋅s^−1^ [7]. With known atomic densities and experimental PT-rate value for different ChG (Table 1), this estimation gives the defect concentration close to ~5⋅10^16^ cm^−3^. It means that under a condition of full identity to void occupation for both annihilating positrons and embedded RE ions, the effect of RE doping can be detected at very low concentrations (reaching only tens of wppm). That is why the PAL spectroscopy can be successfully applied to study RE doping in glassy substances, where conventional atomic-sensitive probes such as X-ray, electron, or neutron diffraction are ineffective because of under-margin content of embedded RE ions, which is typically beyond reliably detectable limits of these methods.

## Conclusions

Atomic-deficient evolution of glassy arsenic selenides is traced in subsequent nanostructurization stages ensuring their RE-doping functionality, the positron annihilation lifetime spectroscopy being employed to parameterize free-volume positron trapping sites within known two-state trapping model. The most efficient positron traps in parent As_2_Se_3_ glass are imagined as voids with character free volumes of ~90 Å^3^ formed in network of corner-shared AsSe_3/2_ pyramids, composed of atomic-accessible geometrical holes (void cores) arrested by surrounding atomic-inaccessible Se-based bond-free solid angles (void shell). Under Ga-codoping in Ga_2_(As_0.40_Se_0.60_)_98_ glass, these voids grow in size, being essentially modified by their environment to become preferentially negative, thus serving as precursors for charge-compensating incorporation of electrically active rare-earth ions. Vitreous state stabilizing modification with Sb additives reduces outer free-volume shell of positron trapping sites mainly due to shielding effect from more metallic environment. The finalizing nanostructurization under Pr^3+^-doping (500 wppm) in Ga_2_(As_0.28_Sb_0.12_Se_0.60_)_98_ glass is explained in terms of competitive contribution of changed occupancy sites available for both rare-earth ions and positrons.

## Abbreviations

BFSA: Bond-free solid angles
ChG: Chalcogenide glasses
FVE: Free-volume elements
PAL: Positron annihilation lifetime
PT: Positron trapping
RE: Rare-earth
